# In Situ Study on Ni–Mo Stability in a Water‐Splitting Device: Effect of Catalyst Substrate and Electric Potential

**DOI:** 10.1002/cssc.202000678

**Published:** 2020-05-12

**Authors:** Jochem H. J. Wijten, Laurens D. B. Mandemaker, Tess C. van Eeden, Jeroen E. Dubbeld, Bert M. Weckhuysen

**Affiliations:** ^1^ Inorganic Chemistry and Catalysis Debye Institute for Nanomaterials Science Utrecht University Universiteitsweg 99 3584 CG Utrecht The Netherlands

**Keywords:** alloys, electrocatalysis, hydrogen evolution reaction, supported catalysts, water splitting

## Abstract

Nickel–molybdenum (Ni–Mo) alloys are well studied as highly effective electrocatalyst cathodes for water splitting. Understanding deactivation pathways is a key to improving the performance of these catalysts. In this study, in situ characterization by UV/Vis spectroscopy and AFM of the morphology and Mo leaching of an Ni–Mo electrocatalyst was performed with the goal of understanding the stability and related Mo leaching mechanism. Switching the potential towards higher overpotentials results in a nonlinear change in Mo leaching. Multiple processes are proposed to take place, such as a decrease in the extent of Mo oxidation at the cathode induced by more strongly reducing potentials, while simultaneously the increase in the local pH at the cathode due to the hydrogen evolution reaction causes more Mo leaching. The change in capacitance of these materials depends strongly on the change in surface composition and not only on the surface area. In situ UV/Vis spectroscopy showed that Mo leaching is a continuous process over the course of 4 h of operation. Finally, the material was deposited on different substrates and the effect on Ni–Mo stability was studied. The substrate has a significant, albeit complex, influence on the stability and activity of Ni–Mo cathodes. In terms of stability in 1 m KOH, Ni–Mo was found to be best deposited on stainless steel substrates operated at low overpotentials, on which it showed nearly no change in capacitance and exhibited low Mo leaching.

## Graphical abstract

**Fulfilled potential**: Ni–Mo is studied as electrocatalyst for the hydrogen evolution reaction. The effect of four substrate materials on its stability is investigated. Furthermore, the effect of applied voltage and current is studied to explore how intermittent electricity results in electrode destabilization. The least Mo leaching is found at low overpotentials with steel as substrate.

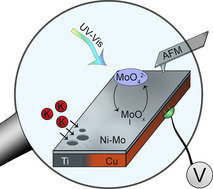

## Introduction

Electrocatalyst materials are widely studied owing to their potential use in the future world energy scheme. Awareness in society of the need for renewable energy sources is growing from both an environmental and economic point of view.^1^, ^2^, ^3^, ^4^, ^5^ However, most of the current processes for renewable energy harvesting yield electricity. This electricity must be stored to overcome the intermittency of these renewable sources, as we cannot control when the wind blows or the sun shines. One of the possible energy storage pathways is to form highly energetic chemical bonds, such as that in hydrogen. Here, electrocatalysts come into play, which allow water to be split into hydrogen and oxygen by using electricity. To allow for an economically and environmentally healthy transition to such a renewable energy system, however, stable and noble‐metal‐free electrocatalysts are required.^1^, ^2^, ^3^, ^4^, ^5^ The stability of electrocatalysts is often tested by quickly cycling between two set potentials for thousand(s) of times.^1^, ^2^ This is done to simulate the change of potential, which is a result of using intermittent electricity from, for example, solar cells and windmills.^1^, ^3^, ^4^, ^5^ In most cases, this cycling shows that electrocatalysts are indeed much less stable when cycling than at constant potential.^1^ For example, it was found for Ni–Fe–S electrodes that high potentials result in oxide formation, which in turn is a pathway to catalyst degradation.^6^, ^7^, ^8^, ^9^, ^10^ This could effectively be halted by using lower concentrations of hydroxide ions at higher potentials.

**Scheme 1:**
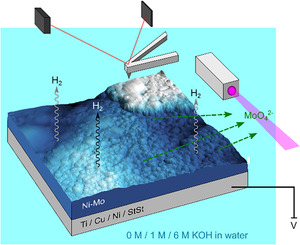
Schematic overview of the approach employed to test Ni–Mo on different substrates. Mo leaching is probed by in situ UV/Vis spectroscopy and AFM at different potentials to reveal the influence of potential on the leaching rate.

Herein, we discuss the effect of potential and the choice of catalyst substrate on the stability of Ni–Mo electrode materials, as is schematically shown in Scheme 1.^11^ The effects of intermittency were studied by applying different constant currents and potentials, with the aim of knowing more about the mechanism of destabilization. By utilizing in situ UV/Vis spectroscopy,^12^, ^13^, ^14^ in situ AFM,^15^, ^16^, ^17^ scanning electron microscopy combined with energy‐dispersive X‐ray spectroscopy (SEM‐EDX), and inductively coupled plasma atomic emission spectroscopy (ICP‐AES), the degree of Mo leaching was determined. Mo is known to be leached from Ni–Mo under the conditions of the hydrogen evolution reaction (HER) as MoO_4_^2−^,^11^, ^18^ and can furthermore be forcibly leached by applying oxidizing potentials for sample preparation.^19^ We also establish the use of in situ measurements for studying operating Ni–Mo electrocatalysts.^11^, ^18^ Building on the knowledge gained previously,^11^ we show the limits for the stability of Ni–Mo and find that the stability is the highest at intermediate potentials. We also explain how the potential/current influences multiple parameters that contribute to the overall observed stability.^20^, ^21^ Furthermore, we studied the stability as a function of substrate type, by utilizing different supports.^22^, ^23^, ^24^, ^25^ Aside from Ti as catalyst substrate, which we used before,^11^ we also studied Ni, since it likely supports metallic growth of Ni–Mo, as well as Cu and stainless steel, which are cheaper materials and thus are likely more attractive for upscaling of new electrodes.

## Results and Discussion

### Effects of electric potential on Ni–Mo/Ti

**Figure 1:**
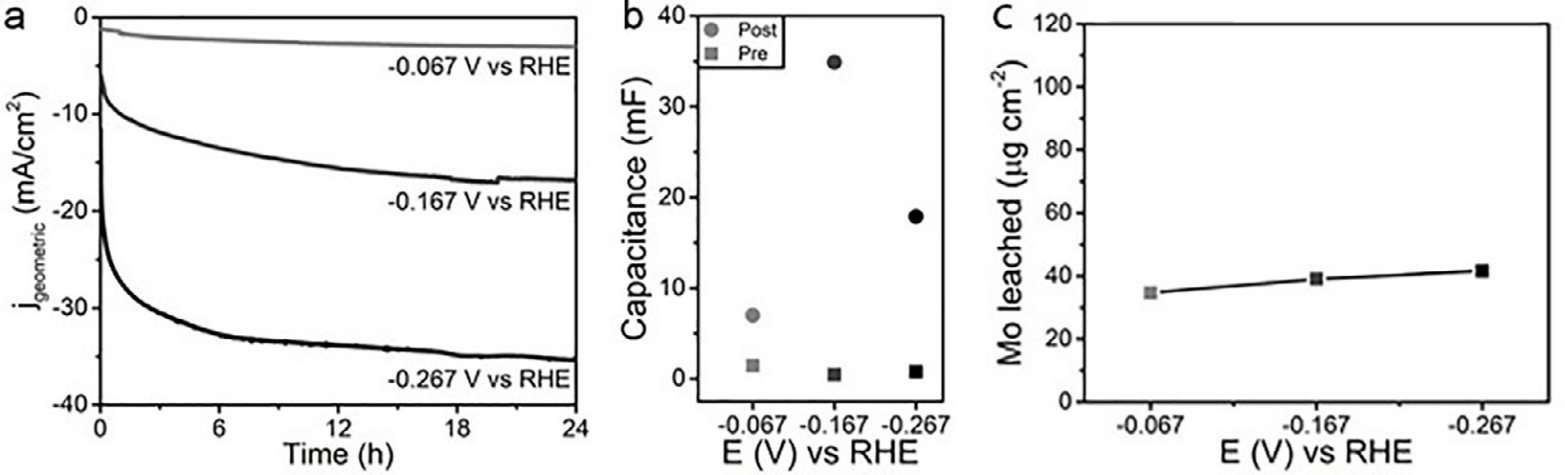
a) Chronoamperometry of Ni–Mo/Ti in 1 m KOH showing the evolution of the current density at different applied potentials. b) Capacitance values found by double‐layer capacitance measurements before (squares) and after (circles) catalysis. c) Amount of Mo leached into the electrolyte per square centimeter of electrode as a function of applied potential after 24 h of chronoamperometric catalysis on Ni–Mo/Ti samples, as probed by ICP‐AES.

To study the effect of the electric potential on the stability of Ni–Mo, Ni–Mo/Ti samples were used to establish a comparison with our previous work.^11^ Figure 1 a shows the chronoamperometric curves for a time span of 24 h. For these curves the potential was at a set value of −0.067, −0.167, or −0.267 V versus RHE and thus the current varies over time. Similar to previous results, activation behavior was observed during the first hour of operation.^11^ As expected, higher potentials resulted in higher currents. More interestingly, for Ni–Mo/Ti it was found that the relative increase in surface capacitance is largest at the intermediate potential of −0.167 V versus RHE (Figure 1 b).

On the contrary, we observed that the surface morphology according to SEM changed most for the sample that was run at −0.067 V (Supporting Information Figure S1). Meanwhile, for the two higher tested potentials, no changes were observed after 24 h. EDX showed that the change in Ni/Mo ratio follows the same pattern as the surface capacitance (Supporting Information, Table S1). Furthermore, it showed that K^+^ infiltration on/into the −0.067 V sample was more significant than for the other two samples. Surprisingly, ICP‐AES (Figure 1 c) showed that Mo leaching simply increases slightly with increasing potential, that is, a more negative potential results in a slightly higher degree of leaching from Ni–Mo/Ti.

The data presented above show that multiple processes take place at the same time, as was already suggested in our previous work.^11^ Herein, we tested, among others, the role of the cation in the leaching of Mo. We found that K^+^ is not adsorbed into the catalyst material itself^11^ but does play a role in the changes that Ni–Mo undergoes during catalysis and is found in/on the material after catalysis. Furthermore, it is likely from the data presented herein that the differences in morphology observed by SEM are correlated to K^+^ infiltration. The exact reason beyond the formation of this sheetlike morphology cannot be stated from the current data. We cannot conclude whether the presence of K^+^ is the cause or a result of the change in morphology. The samples give no appreciable XRD signal; thus, the phase of the material is not known. From our previous work,^11^ we know that vacancy formation by Mo leaching has different energies on different surface facets, which could give rise to this observed morphology change.

Meanwhile, the Ni/Mo ratio observed by SEM‐EDX can clearly not directly be linked to the observed leaching by ICP‐AES. This can be explained by surface segregation, as was already discussed in depth in previous studies.^11^, ^26^, ^27^ Movement of Ni and Mo through the bulk of the system results in higher Ni/Mo ratios near the surface, which lowers the observed Mo content due to the limited penetration depth of the electron beam. Since the capacitance follows the Ni/Mo trend observed by SEM‐EDX (increases of 1.16, 2.87, and 1.47, respectively, as a function of potential) and not the leaching trend observed by ICP‐AES, it can be concluded that the change in Ni/Mo ratio is the main contributor to the observed changes in capacitance and not the surface area. This underlines previous reports that care should be taken when linking the capacitance to the surface area.^28^, ^29^, ^30^

**Figure 2:**
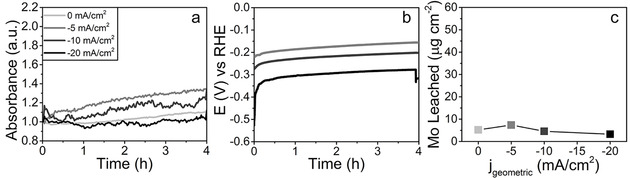
a) Absorbance at 232 nm (MoO_4_^2−^), as probed by transmission UV/Vis spectroscopy and as a function of time. b) Chronopotentiometric curves at −5, −10, and −20 mA cm^−2^. c) Amount of Mo found in the electrolyte after 4 h of catalysis.

Furthermore, the link between capacitance and activity could lead to the hypothesis that the change in activity is mostly due to the surface composition and not to an increase in surface area. The rate of Mo leaching was monitored over time to increase our understanding. Figure 2 a shows in situ UV/Vis spectroscopic data, demonstrating the evolution of the MoO_4_^2−^ peak over time.^11^ The UV/Vis spectra of a representative sample are shown in Figure S2. Note that, from a practical point of view, we used chronopotentiometry (Figure 2 b) and not chronoamperometry, because of bubble formation, which is assumed to be constant during chronopotentiometry. Thus, the possible influence of bubbles, which could cause variable scattering effects in different experiments, is minimized.^31^

Most importantly, as the chronopotentiometric curve starts to level off, there seems to be no observable response yet in the evolution of the MoO_4_^2−^ peak, in further agreement with the above hypothesis that more processes influence the observed activity and hinting that another process is dominant in the time frame of activity. Counterintuitively, we observe that the Mo leaching decreases with increasing current in the UV/Vis spectroscopic experiments. An increase in noise and baseline signal compared to the white and dark spectrum was nevertheless observed due to bubble formation. The leaching changes with varying current, as could be expected, and passes through a maximum at −5 mA cm^−2^. Note that the observed current–potential magnitude cannot be directly compared to the chronoamperometric data in Figure 1 a because different electrochemical cells were used (see Experimental Section) and thus different electrolyte resistances and kinetics are expected.^32^, ^33^ The same trend with maximum leaching at −5 mA cm^−2^ is observed in both the in situ UV/Vis spectroscopic and the ICP‐AES experiments, and this suggests that in situ UV/Vis spectroscopy can indeed be used to monitor Mo leaching in these systems. Our proposed reason behind the observation of the highest Mo leaching at intermediate values is twofold. On the one hand, the HER (2 H_2_O+2 e^−^→2 OH^−^+H_2_) forms one OH^−^ ion locally for each electron consumed in the reaction.^32^ This results in a significantly higher pH directly at the surface. As was already described in our previous work on the mechanism,^11^ the reaction Mo+2 OH^−^+2 H_2_O→MoO_4_^2−^+3 H_2_ results in increased Mo leaching due to OH^−^. On the other hand, the half‐reaction of leaching (Mo+8 OH^−^→MoO_4_^2−^+4 H_2_O+6 e^−^) is oxidative, so thermodynamically it makes sense that the leaching is halted by strongly reducing potentials.

**Figure 3:**
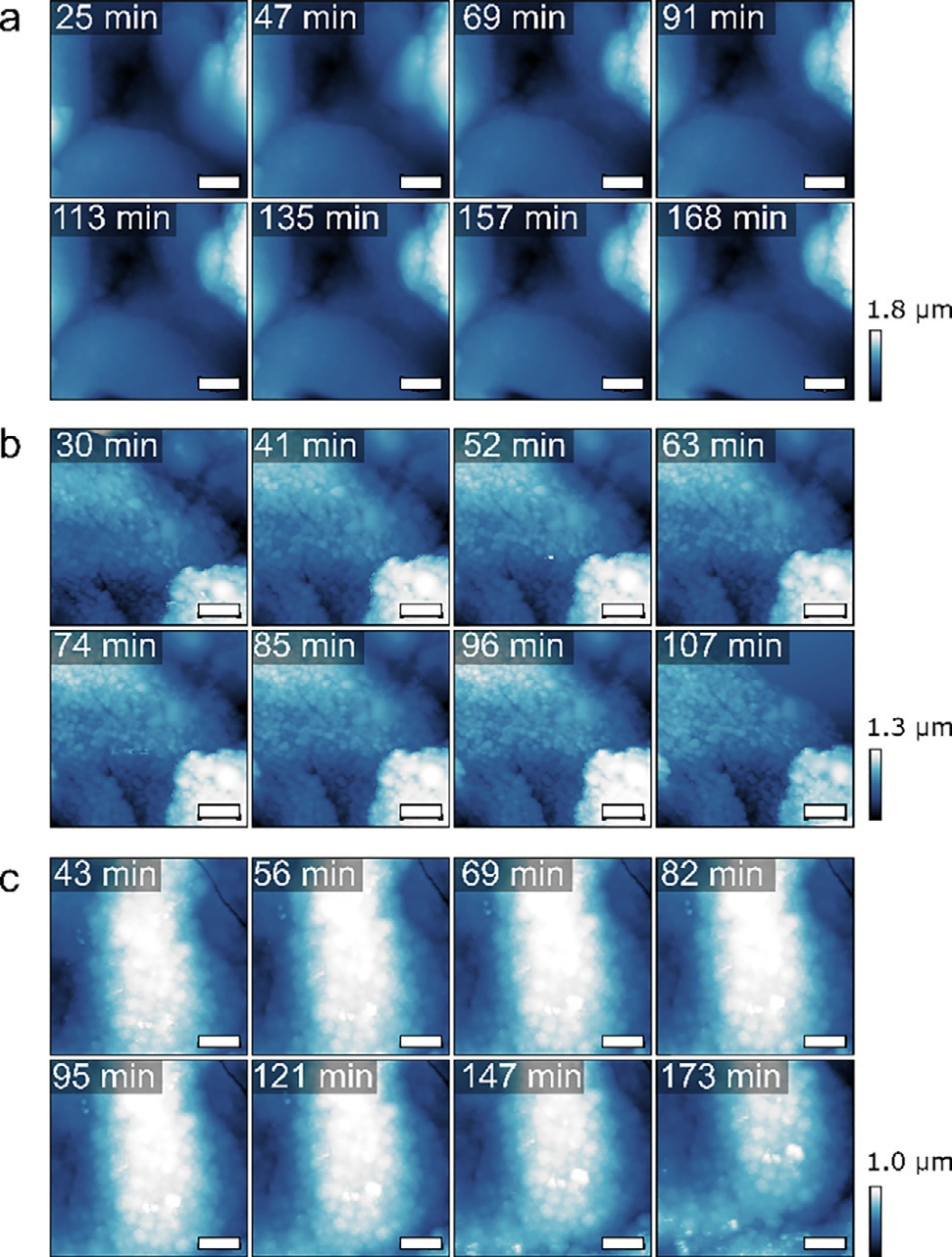
AFM images of Ni–Mo/Ti catalysts measured in situ at different times at a) 0 m, b) 1 m, and c) 6 m KOH. The scale bars are 2 μm.

**Figure 4:**
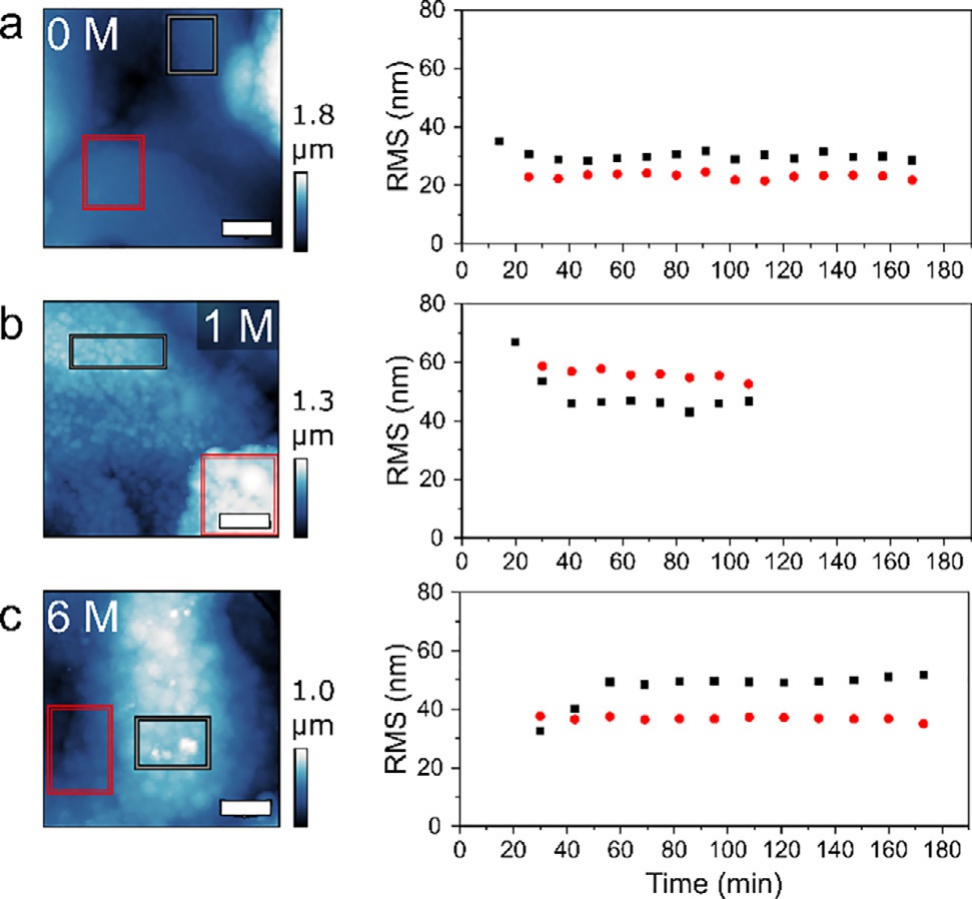
Left: AFM images of the Ni–Mo/Ti catalysts with two selected spots on each material immersed in a) 0 m, b) 1 m, and c) 6 m KOH. Right: progression of absolute RMS as a function of time of these samples at the two selected spots. The scale bars are 2 μm.

In line with our previous work, we observed that the change in morphology (Figure S3) is correlated to the amount of K^+^ found in the material by SEM‐EDX (Table S2). The initial Ni/Mo ratio in these samples was relatively low, but the impact on the performance or observations was minimal. Furthermore, using in situ AFM, we revisited probing of the outer surface area. Although AFM proved unsuitable to visualize the formation of pores in the Ni–Mo electrocatalysts, the evolution of the Ni‐Mo surface can be monitored at the exact same position without influencing the processes by drying or dispersion, which would be the case in an ex situ approach. We studied the samples for KOH concentrations of 0, 1, and 6 m without exposing them to an applied potential. This enables visualization of the catalysts solely in dispersion, as a background check, in which possible leaching effects of the electrochemical work are cancelled out. Hence, to elucidate the role of the surface area in the electrochemical activity, the in situ AFM data were compared to the evolution of the electrochemical curve. Figure 3 shows the micrographs over time for the different concentrations. At first sight, some drift is visible, but the main domains and morphology of the material are preserved. The surface roughness, expressed as root mean square (RMS) values, of the materials is a proper attribute to study leaching effects, as was shown before.^11^ As large or many height contrasts influence the RMS, specific regions of interest (ROIs) were chosen and their RMS plotted over time (Figure 4). The initial RMS values were similar, which highlights that the chosen ROIs can be properly compared. If the background processing of the AFM images is performed for the full micrograph, the drift over time will change the background and hence the resulting RMS in the ROIs. Proper background corrections are described in the Experimental Section. Figure 4 shows that different locations, indicated by the red and black rectangles (Figure 4 a and c) and corresponding plots (Figure 4 b and d respectively) on the sample have different behaviors in terms of RMS over time. It can be expected that these samples, which are relatively rough and morphologically inhomogeneous compared with the typically well‐defined film materials, have different domains with different behaviors. The material forms cauliflower‐like structures made of intergrown spheres. These structures have relatively low RMS values, whereas the RMS measured over several of these intergrowths is relatively high. Using in situ AFM we demonstrate that this is indeed the case and that likely the particulate, rougher features, such as the large grains of intergrowths observed in the black‐marked spot in the 1 and 6 m experiments, are the most sensitive to leaching. Whereas a large grain disappeared in the 1 m experiment, with resulting decrease in the RMS after approximately 40 min, a large grain was more exposed at 6 m and the RMS increased. As these ROIs are constant after the initial change due to the grains, and the other ROIs show hardly any increase or decrease at all, these data suggest that morphological evolution on the cauliflower‐like surface does not occur without applied electrochemistry. This indicates that the Ni–Mo structure is stable for at least several hours in solution if no potential is applied, so that electrical downtime (e.g., during the night if solely solar panels are used) would not result in significant catalyst damage; this is an important property for practical applications. On a side note, we can conclude that AFM can be performed in situ over extended periods of time without any visible tip effects (Si_3_N_4_ tips) under extreme alkaline conditions, as this would also have a large influence on the observed features in Figures 3 and 4.

### Substrate effects on the stability of Ni–Mo

In this work, we studied four substrates: Ti, Ni, Cu, and stainless steel (StSt). Ti was studied as a model substrate that was used previously due to its durability and practically non‐existent HER activity.^34^ Ni and Cu were chosen since they have a face‐centered cubic (FCC) crystal structure,^35^ which is the expected crystal structure for Ni–Mo as well.^36^, ^37^ Finally, StSt was chosen since it is a readily available and durable material.

**Figure 5:**
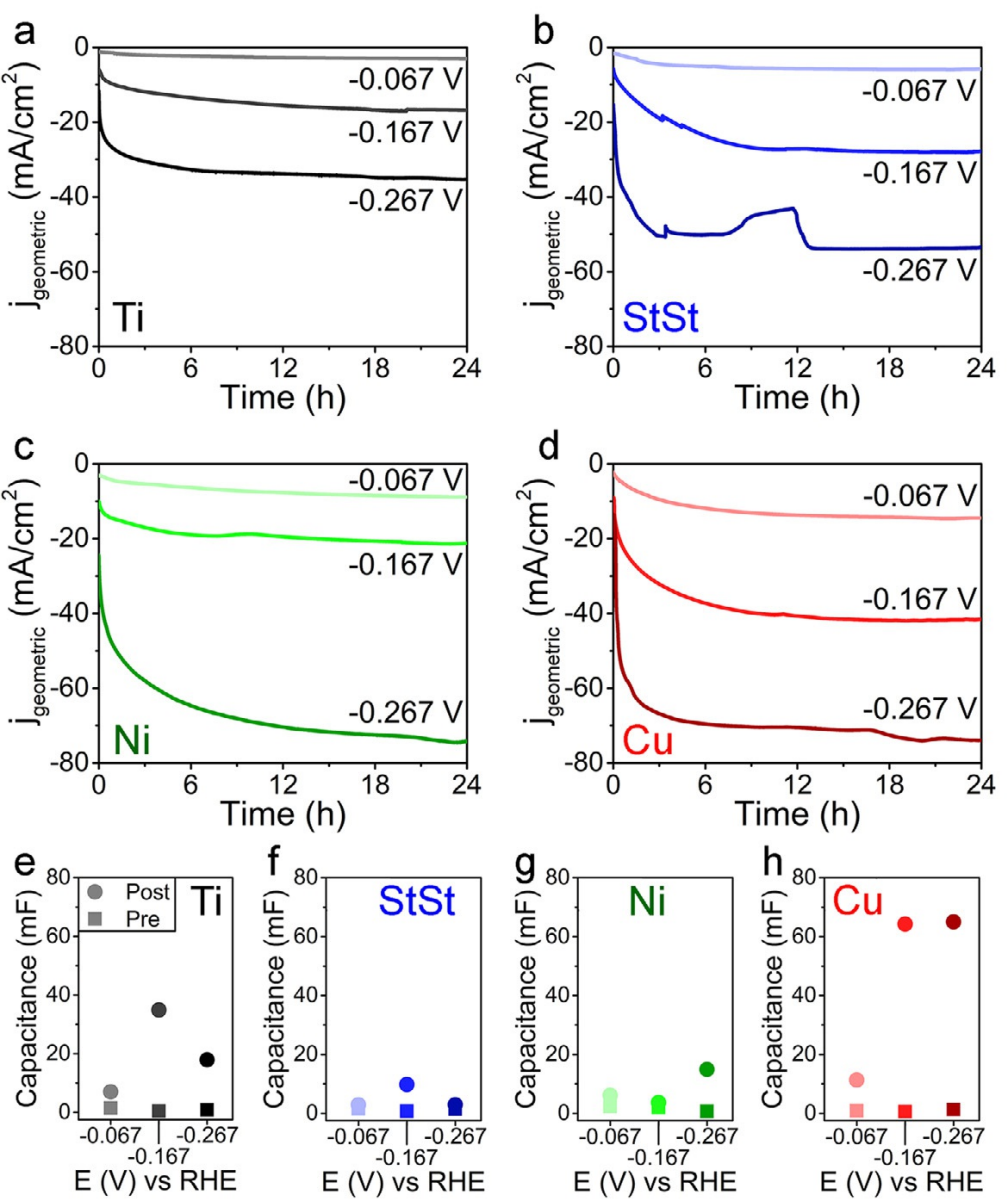
Chronoamperometric curves at −0.067, −0.167, and −0.267 V on a) Ni–Mo/Ti, b) Ni–Mo/StSt, c) Ni–Mo/Ni, and d) Ni–Mo/Cu. The double‐layer capacitance values before (squares) and after (circles) are shown for Ni–Mo on e) Ti, f) StSt, g) Ni, and h) Cu.

As shown in Figure 5 Ni–Mo behaves significantly differently on different substrates. The performance of the materials is strongly dependent on the substrate as well. The activity decreases in the order Cu>Ni>StSt>Ti. In all cases activation over time occurred, which hence can now be safely assumed to be an effect of Ni–Mo and not substrate‐related. Additionally it cannot be an effect of the catalytic activity of the substrates, since it is well known that StSt and Ni are significantly better at HER than Cu. Another interesting observation is that on Cu the activation at higher currents is significantly faster, especially at −0.267 V versus RHE, which suggests that restructuring of the Ni–Mo catalyst occurs faster on Cu.

Meanwhile there are interesting observations when considering the change in capacitance. StSt, like Ti, results in an intermediate potential at which the change in capacitance is most significant. For Ni and Cu, which have FCC structures, higher potentials (within the tested range) result in larger capacitance changes.

Studying the Ni–Mo deposits by SEM (Figures S4–S6 for Ni, Cu, and StSt, respectively) showed similar morphologies. Furthermore, studying these SEM images and comparing them with Figure S1 for Ni–Mo/Ti reveals that the morphologies of these samples behave similarly, whereby the initial spherical, cauliflower‐like structure forms a sharper morphology after catalysis. Note that all micrographs are in focus, and in the cases in which they do not seem to be so, it was due to a lack of contrast in the sample, and in these cases cracks near the premeasured spot were used to achieve optimal focus. All the samples show a similar morphological response to the presence of K^+^ at the sample surface. As shown in Table S3, loss of Mo always occurs from all these samples. The extent of observed leaching/segregation, however, is significantly different and seems to depend on an interplay of potential and substrate.

If we compare the loss of Mo observed with EDX with ICP‐AES (Figure S7) it is clear that the change in EDX signal results from a combination of Mo leaching and surface segregation. Furthermore, no leaching of the substrates themselves was observed by ICP‐AES. The fact that the double‐layer capacitance does not agree takes away any doubt that indeed multiple processes influence the data. The order of magnitude of leaching changes with substrate, and Mo is more stable on average at the tested potentials in the order of Ti>Ni>Cu>StSt. As the morphological shape is not significantly influenced by the substrate, this effect of the substrate is likely electrostatic: the energetics of the reaction Mo+2 OH^−^+2 H_2_O→MoO_4_^2−^+3 H_2_ changes. The shift in optimum potential means that the ratio of effects of local OH^−^ concentration and Mo stabilization is different.

**Figure 6:**
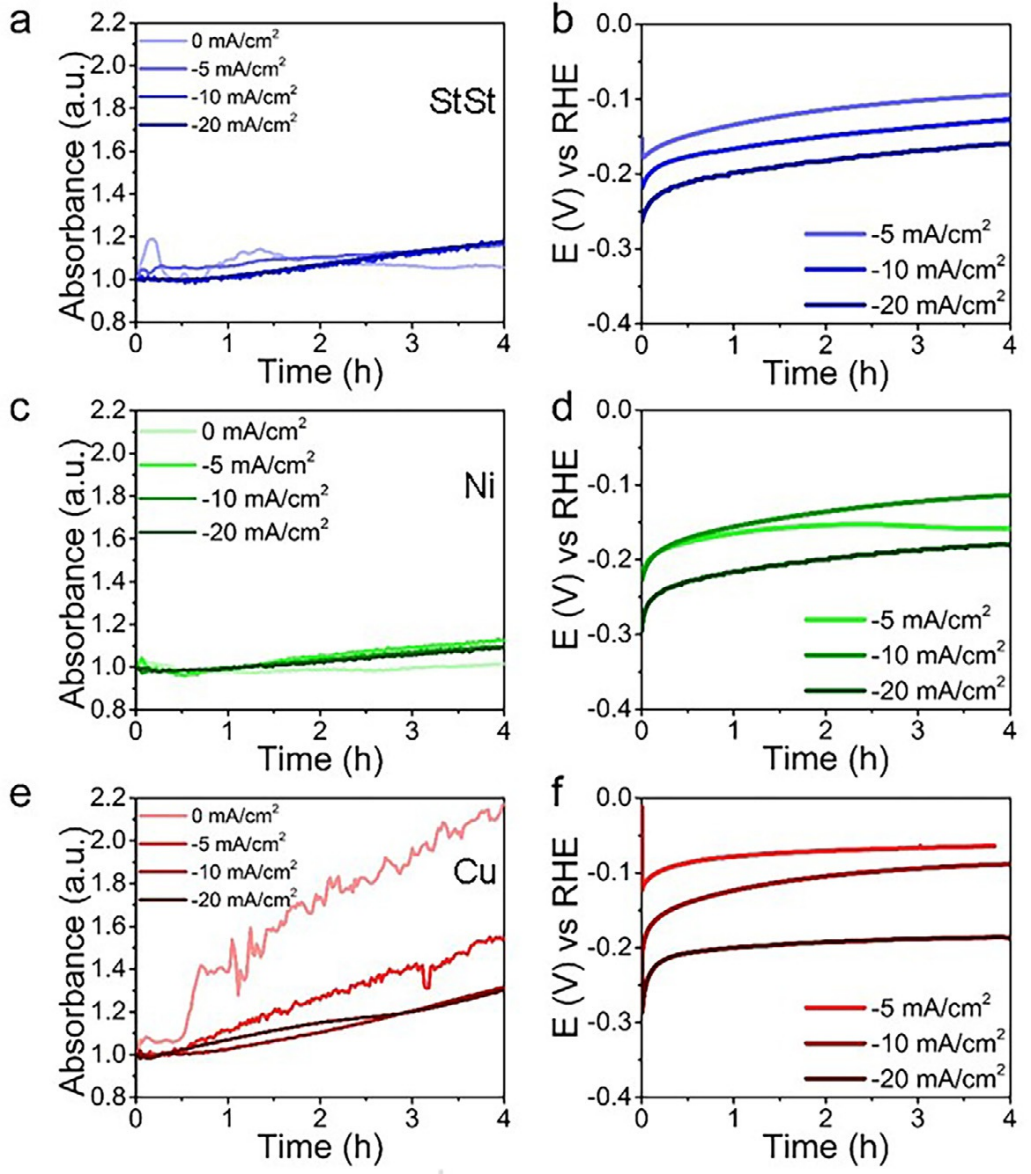
a) UV/Vis spectroscopic intensity measured at 232 nm over time and b) the corresponding chronopotentiometric curves on Ni–Mo/StSt. c) UV/Vis signal at 232 nm over time and d) the corresponding chronopotentiometric curves on Ni–Mo/Ni. e) The UV/Vis signal intensity at 232 nm over time and f) the corresponding chronopotentiometric curves on Ni–Mo/Cu.

To study possible changes in the leaching pattern due to the substrate, in situ UV/Vis spectroscopy was performed (Figure 6). As was the case with Ni–Mo/Ti, Ni, Cu, and StSt show a fairly constant leaching rate in at least the first 4 h. The rates, however, are vastly different, and StSt and Ni perform best in terms of leaching suppression at set currents, closely followed by Ti (Figure 2 a). On Cu, however, we observed significantly higher MoO_4_^2−^ signals compared with the other substrates, especially when no current was applied. Considering the faster restructuring observed in Figure 5 it can be assumed that using Cu as a substrate for Ni–Mo forces the equilibrium of Mo leaching towards MoO_4_^2−^, and thus results in higher leaching rates. The ICP‐AES data of the spent electrolytes of these samples can be found in Figure S8.

This suggests that re‐adsorption or even re‐deposition of Mo likely takes place during the tests, which could also explain the morphological changes observed by SEM, as the deposition conditions are different to those used in the material synthesis.^38^ This would also explain our previous observation by depth‐profile XPS^11^ that the direct surface concentration of Mo is higher than that in in the bulk. This re‐deposition effect is also seen with EDX (Table S4), as the change in Ni/Mo ratio disagrees with the ICP‐AES data owing to the inhomogeneity in depth of the material. Furthermore, in some cases we even observed decreasing Ni/Mo ratio, even though for none of the tested samples was Ni was found to be leached. This effect is mostly seen for the Cu substrate and might be related to the fact that the in situ UV/Vis intensity related to MoO_4_^2−^ is high, which suggests a lot of MoO_4_^2−^ dissolution.

## Conclusions

The stability of the Ni–Mo system as electrocatalyst cathode for water splitting was explored. The effect of K^+^ inclusion could be ascribed to morphology changes, and both Mo leaching and surface segregation were observed. The change in double‐layer capacitance was found to be most sensitive to the change in Ni/Mo ratio on the surface and not surface roughening. It was shown that Mo leaching has a constant rate in the first 4 h by in situ experiments.

On the one hand, the effect of potential was studied, and it was found to influence the stability on several levels. Since there is an optimum potential for the Ni–Mo/Ti system, the potential has both stabilizing and destabilizing effects on the material. These were ascribed to the reducing potential hampering Mo oxidation to Mo^6+^ (in the form of MoO_4_^2−^), whereas more strongly reducing potentials also lead to more HER and thus higher local pH values (OH^−^ concentrations), which increase Mo leaching, as seen in the leaching mechanism Mo+2 OH^−^+2 H_2_O→MoO_4_^2−^+3 H_2_. Additionally, no potential resulted in no surface change, as found by in situ AFM, which is proof of the practical potential of these materials as electrocatalysts, as they are only deactivated during electrocatalysis and not during inactive periods in solution.

On the other hand, we have studied the effect of the support material on the leaching and activity of the electrocatalyst. The stability of Ni–Mo was studied on Ti, Cu, Ni and StSt. The substrate has a significant influence on the behavior of the deposited Ni–Mo and potentially other electrocatalysts as well. Regarding activity, we found the highest currents for Ni–Mo/Cu and Ni–Mo/Ni followed by Ni–Mo/StSt and finally Ni–Mo/Ti.

According to UV/Vis spectroscopy slow buildup of MoO_4_^2−^ occurs near the electrode surface in each system. This is in disagreement with the ICP‐AES data and led us to suggest that MoO_4_^2−^ is not only leached but also re‐adsorbed, as was further suggested by the morphological changes found with SEM‐EDX.

The best stability for Ni–Mo was found at −0.067 V versus RHE for Ni–Mo/StSt, which exhibited the least Mo leaching after 24 h and a very small change in capacitance. Thus, by using Ni–Mo as an example, we have shown that the choice of electrode substrate for electrodeposited electrocatalysts can have a significant impact on both catalyst stability and performance.

## Experimental Section

**Chemicals and materials**: All materials were used as received without further purification. NiSO_4_ **⋅**6 H_2_O (ReagentPlus, >99 % pure), NaMoO_4_ **⋅**2 H_2_O (ACS reagent, >99 % pure), KOH (ACS reagent, >85 % pure), and Na_3_C_6_H_5_O_7_ **⋅**2 H_2_O (sodium citrate, ACS reagent, >99 % pure) were received from Sigma Aldrich. NH_3_ (28–30 %, ACS reagent, Ph. Eur. for analysis) was obtained from Emsure. In all experiments, deionized water was used.

**Electrodeposition**: Ti stubs (99.99+ %, Goodfellow), Ni stubs (99.99+ %, Goodfellow), Cu stubs (99.99+ %, Goodfellow), and StSt (AISI 316L, 69 % Fe, 18 % Cr, 10 % Ni, 3 % Mo, Goodfellow) were machined into round substrates of similar shape to SEM stubs with a surface area of 1.257 cm^2^. The stubs were fixed in three‐electrode cells for electrodeposition after being polished with SiC paper with increasing grit (500, 1200, 4000), and cleaned by sonication in three steps (15 min each in 1:1 ethanol/acetone, 2 m HNO_3_, and deionized water).^11^ Galvanostatic electrodeposition was performed with an Ivium Compactstat at a current of −100 mA for 1200 s while stirring at 400 rpm. A Pt‐mesh electrode (Mateck, 99.9+ %) was used as counter electrode, and no Pt leaching was observed.^11^ A 3 m KCl BASi Ag/AgCl electrode was used as reference electrode.

The used plating baths contained 0.3 m NiSO_4_, 0.2 m Na_2_MoO_4_, and 0.3 m Na_3_C_6_H_5_O_7_ in 100 mL of deionized water. To these solutions 10 mL of aqueous NH_3_ was added to achieve a pH of 9.2. Separate plating baths were used for each substrate type to prevent cross‐contamination of the substrates. Prior to deposition, the plating bath was deoxygenized by purging with Ar (5.0) to at a flow rate of 20 mL min^−1^ for at least 15 min. The average Ni/Mo ratio (from SEM‐EDX) of the materials after synthesis was 2.73±0.72 (Ni–Mo/Ti), 1.78±0.69 (Ni–Mo/StSt), 13.47±9.7 (Ni–Mo/Ni), and 2.35±0.28 (Ni–Mo/Cu). The error for Ni–Mo/Ni is large due to the change in substrate contribution to the signal as a result of changing deposition thickness.

**Electrochemical characterization**: The samples were loaded in three‐electrode cells with a Pt‐mesh counter electrode and a 3 m Ag/AgCl [Metrohm, shielded, −0.207 V vs. SHE, which is −1.033 V vs. RHE (pH 14)] reference electrode. 1 m KOH was used as electrolyte at pH 14. First, double‐layer capacitance was measured between −0.7 V and −0.9 V versus Ag/AgCl at 100, 200, 300, 400, and 500 mV s^−1^ for three cycles each. Following this, a linear sweep was performed at 50 mV s^−1^ from −1 V to −1.3 V versus Ag/AgCl to confirm that no polluting electrochemical processes take place (data not shown). Finally, chronoamperometry was performed for 24 h at −1.1, −1.2, and −1.3 V versus Ag/AgCl (−0.067, −0.167, and −0.267 V vs. RHE). The linear‐sweep and double‐layer capacitance measurements were performed again afterwards. Double‐layer capacitance values were obtained by taking the difference between the forward and backward currents of the third scan. Data were averaged between 0.795 and 0.805 V for each point. The resulting double‐layer thickness was then plotted versus the scan rate (at 100, 200, 300, 400, and 500 mV s^−1^). The linear fit of these points yields the capacitance.

During the in situ UV/Vis spectroscopic experiments, the samples were loaded in a three‐electrode Suprasil Quartz cell (Figure S9). Chronopotentiometry was performed for 4 h at −5, −10, and −20 mA cm^−2^. The potentiostat was not used for the 0 mA cm^−2^ measurements. Neither linear‐sweep nor capacitance measurements were performed in these cases. A Pt wire (Mateck, 99.9+ %) was used as a counter electrode, and a 3 m BASi Ag/AgCl reference electrode was used.

**In situ UV/Vis spectroscopy**: In situ UV/Vis spectroscopy was performed with a PerkinElmer Lambda 950s spectrometer. Measurements were done from 600 to 200 nm with 4 nm intervals, which allows for 180 measurements during 4 h. A D_2_ lamp and a W lamp were used, and the switch was set at 319.2 nm. Measurements were done in transmission mode and a blank was taken prior to each measurement. Dense black cloth was effectively used to block incoming ambient light through the small opening left by the potentiostat cable, tested by measurements with and without the cloth and with and without the cable (data not shown). A long measurement range was used in each experiment as a control for possible Ni, Cu, Cr, Fe, or Ti leaching. Background subtraction was performed based on the first spectrum in the range of 600–500 nm. The variation in background between the 180 measurements per sample was <1 %, that is, there was no significant impact of scattering by the formed hydrogen bubbles. The MoO_4_^2−^ peak was analyzed by plotting the intensity at the rising part of the band (232 nm), since O_2_ UV absorption (ozone formation) started at lower wavelengths and resulted in significant interference.

**In situ AFM**: In situ liquid‐phase AFM measurements were performed with a Bruker Multimode microscope by using SNL‐10A silicon nitride cantilevers (*F*=0.175 N m^−1^) in PeakForce tapping mode. The moment of sample immersion was set as *t*=0, then the AFM was optimized to measure a 10×10 μm^2^ spot with a frequency of 0.8 Hz, resulting in an image every 11 min. The data were post‐processed with Gwyddion. First, the images were flattened by using a plane background subtraction and a trimmed median of differences row alignment. The resulting images were used in Figure 3. Subsequently, ROIs that were found throughout the majority of time frames were selected. These areas were cropped, flattened in a similar manner as described above, and then used to plot the roughness (RMS, from “Statistical Functions”). The crop/flattening was essential, as drift highly influences the general background plane in the full images (as can be seen from the two different displayed time snapshots),and different RMS values over time result when they are not selectively cropped (Figure S10). The black crosses represent the RMS of the selected area plotted after flattening was performed over the full images. The red crosses represent the RMS of the selected area plotted after cropping the selection and repeating the flattening steps, and fundamentally represents the roughness of the selected surface.

**Structural characterization**: ICP‐AES was performed with an Optima 8300 instrument from PerkinElmer. Electrolytes were decreased in pH by adding 1 mL of 65 % HNO_3_ per 10 mL electrolyte, resulting in approximately 2 % HNO_3_. Ni (231.604 nm), K (766.491 nm), Na (589.592 nm), Ti (334.187 nm), Cr (205.560 nm), Cu (327.393 nm), Fe (259.939 nm), Pt (214.423 nm), and Mo (202.095 nm) were then measured. SEM‐EDX was performed with an FEI Helios NanoLab 600 DualBeam instrument with an Oxford Instruments Silicon Drift Detector X‐Max energy‐dispersive spectrometer. EDX mapping was performed with an electron beam of 15 kV and 0.8 nA. All SEM imaging was done with secondary electrons at 15 kV and 0.8 nA. Wide images were first shot to ensure the larger magnifications were taken at representative areas of the material (data not shown for brevity).

## Conflict of interest

*The authors declare no conflict of interest*.

## Supporting information

As a service to our authors and readers, this journal provides supporting information supplied by the authors. Such materials are peer reviewed and may be re‐organized for online delivery, but are not copy‐edited or typeset. Technical support issues arising from supporting information (other than missing files) should be addressed to the authors.

Supplementary
