# Supplementary material for: In Situ Study on Ni–Mo Stability in a Water‐Splitting Device: Effect of Catalyst Substrate and Electric Potential
Source: ChemSusChem. 2020 May 12;13(12):3172–9. doi: 10.1002/cssc.202000678 (PMC7317784; doi:10.1002/cssc.202000678)
Supplement: Supplementary file 1 — Supplementary [file CSSC-13-3172-s001.pdf]

ChemSusChem

Supporting Information

In Situ Study on Ni–Mo Stability in a Water-Splitting Device: Effect of Catalyst Substrate and Electric Potential

Jochem H. J. Wijten, Laurens D. B. Mandemaker, Tess C. van Eeden, Jeroen E. Dubbeld, and Bert M. Weckhuysen*[a]

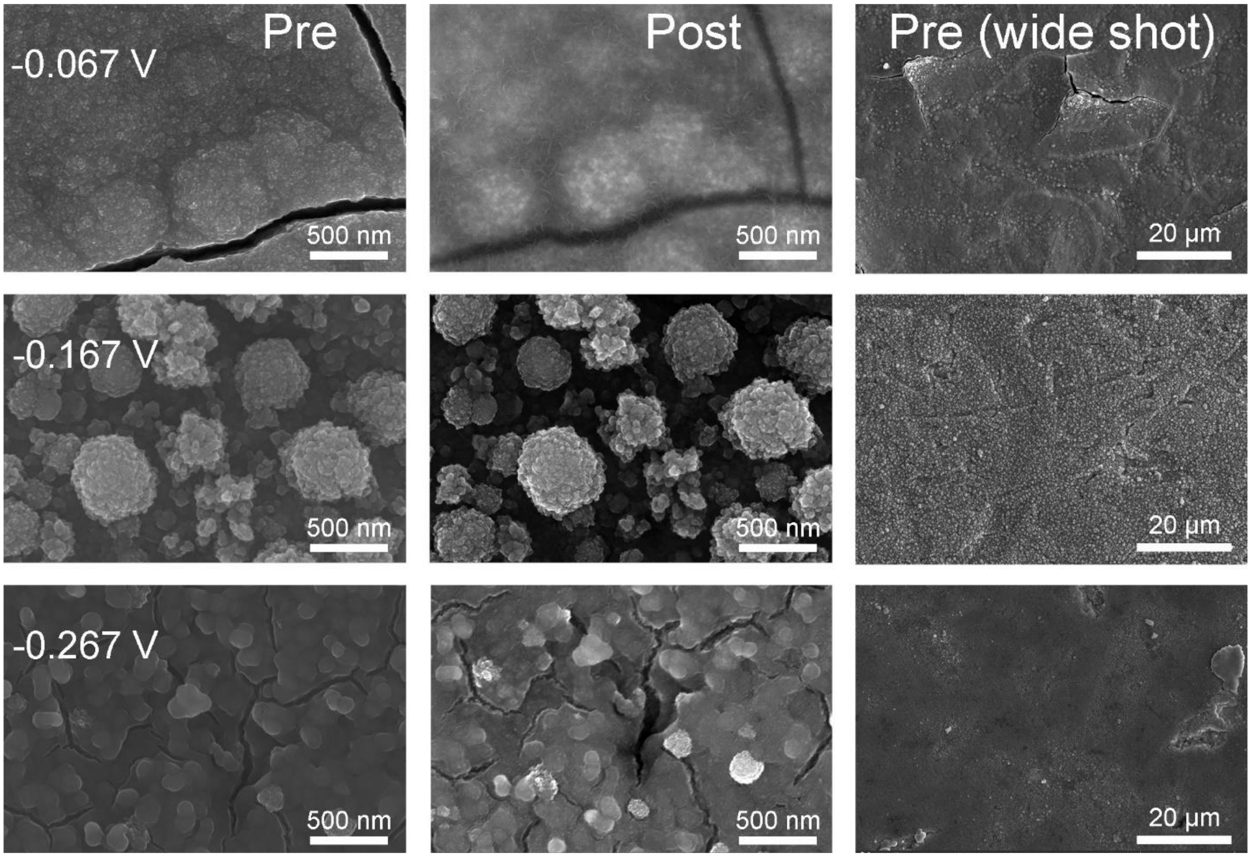

Figure S1. SEM micrographs of Ni-Mo/Ti before (left) and after (middle) chrono-amperometric catalysis for 24 h in 1 M KOH at different potentials. At the right are SEM micrographs of the same sample before catalysis comparing them on a larger scale, no differences are visible after catalysis on this scale.

Table S1. Atomic ratios as found by SEM-EDX before (regular) and after (bold) 24 h of chrono-amperometry. Only Ni, Mo, Na and K are considered and thus add up to 100 at%. Other elements that were observed are (adventitious) C, O, Ti, and Si, the latter sometimes being present as SiC particles that had been stuck in groves left by the sanding paper. The last column shows the ratio of Ni/Mo.

	Ni (at%)	Mo (at%)	Na (at%)	K (at%)	Ni/Mo ratio
Ni-Mo/Ti -0.067 V	64.8	31.4	3.8	-	2.06
**Ni-Mo/Ti -0.067 V**	**68.8**	**28.9**	**-**	**2.4**	**2.38**
Ni-Mo/Ti -0.167 V	73.2	22.8	3.9	-	3.21
**Ni-Mo/Ti -0.167 V**	**88.5**	**9.6**	**1.0**	**1.0**	**9.22**
Ni-Mo/Ti -0.267 V	51.0	39.0	10.0	0	1.31
**Ni-Mo/Ti -0.267 V**	**64.9**	**33.8**	**-**	**1.4**	**1.92**

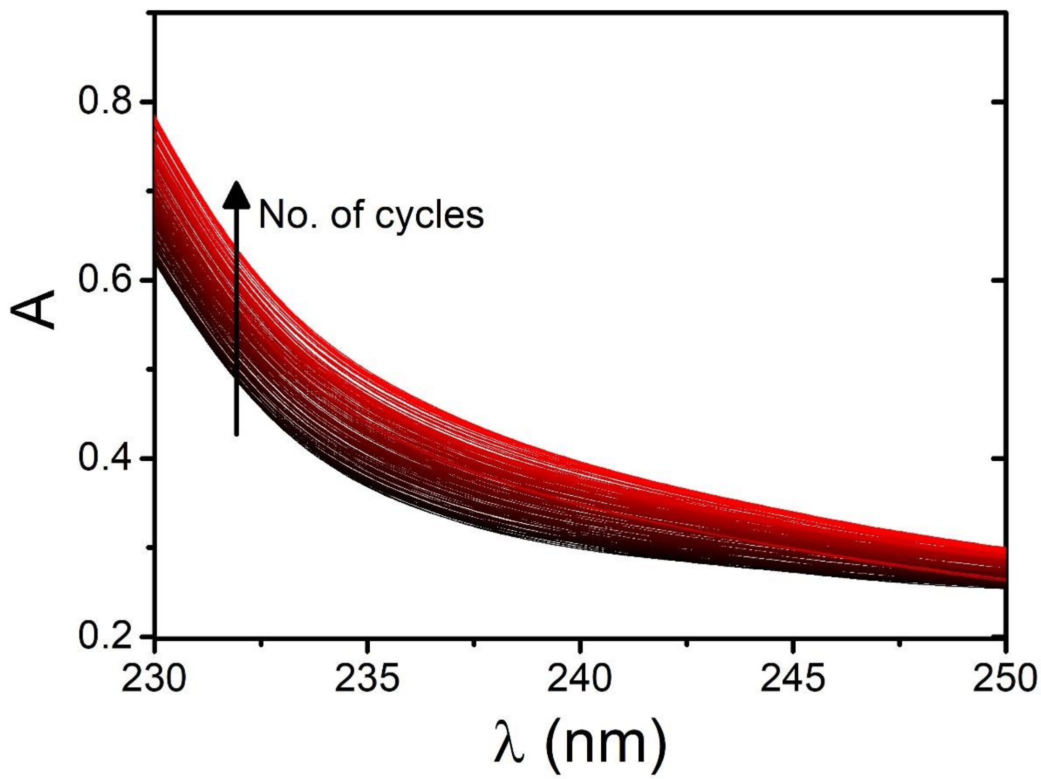

Figure S2. UV-Vis Spectra of Ni-Mo/Cu at -5 mA/cm^2 showing how the spectra evolved in the MoO_4^{2-} shoulder at 232 nm.

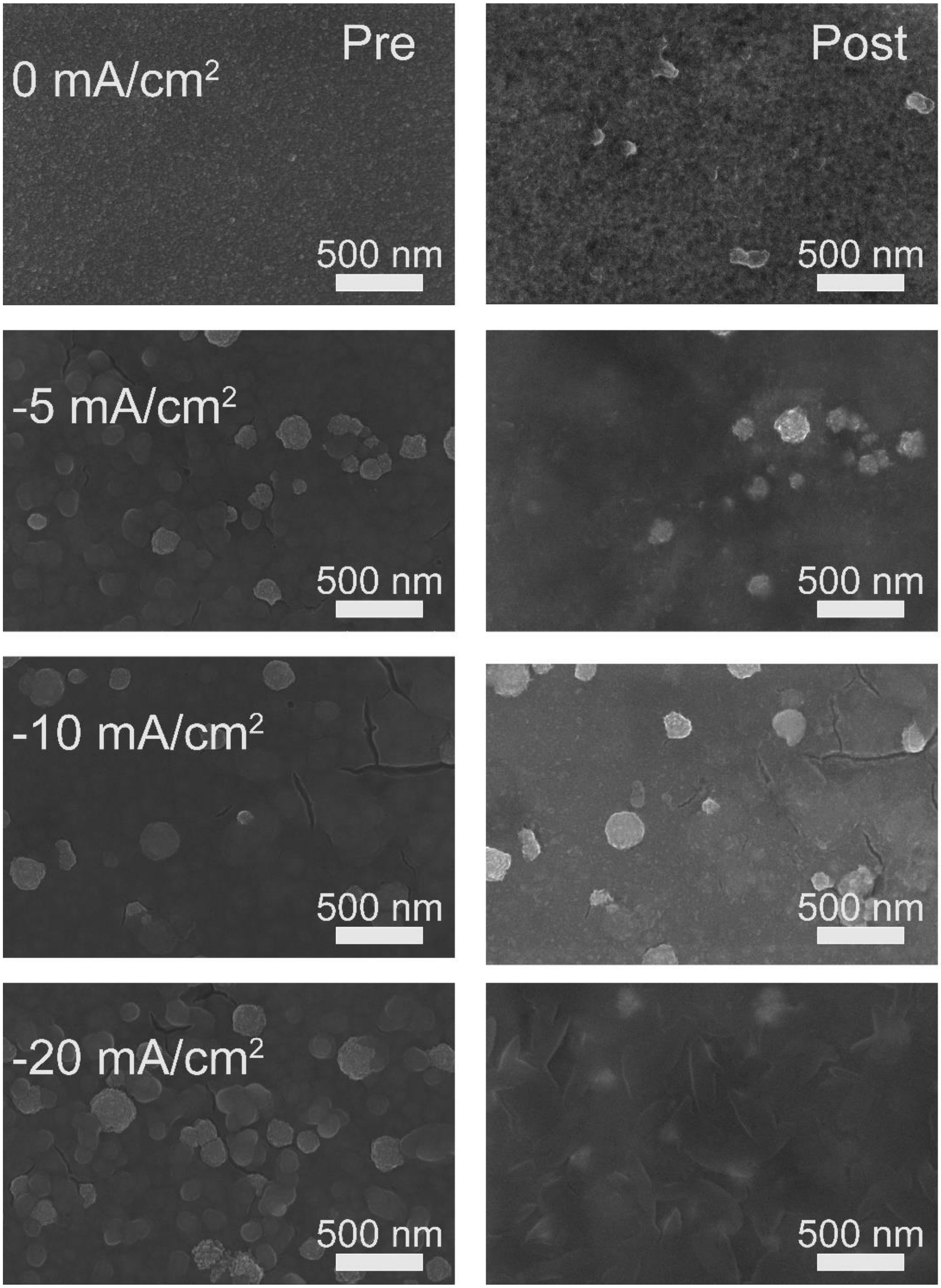

Figure S3. SEM micrographs of Ni-Mo/Ti before (left) and after (right) 4 h of chronopotentiometry at 0, -5, -10 and -20 mA/cm^2, respectively.

Table S2. Atomic ratios as found by SEM-EDX before (regular) and after (bold) the *in situ* UV-Vis spectroscopy experiments. Only Ni, Mo, Na and K are considered and thus add up to 100 at%. Other elements that were observed are (adventitious) C, O, Ti, and Si, the latter sometimes being present as SiC particles that had been stuck in groves left by the sanding paper. The last column shows the ratio of Ni/Mo.

	Ni (at%)	Mo (at%)	Na (at%)	K (at%)	Ni/Mo ratio
Ni-Mo/Ti 0 mA/cm^2	67.8	30.1	2.1	-	2.25
**Ni-Mo/Ti 0 mA/cm^2**	**65.4**	**32.0**	**2.2**	**0.5**	**2.04**
Ni-Mo/Ti -5 mA/cm^2	47.8	40.2	12.0	-	1.19
**Ni-Mo/Ti -5 mA/cm^2**	**39.4**	**24.3**	**8.7**	**27.6**	**1.62**
Ni-Mo/Ti -10 mA/cm^2	44.6	40.6	14.8	-	1.10
**Ni-Mo/Ti -10 mA/cm^2**	**52.1**	**34.8**	**5.0**	**8.1**	**1.50**
Ni-Mo/Ti -20 mA/cm^2	50.4	38.0	11.6	-	1.33
**Ni-Mo/Ti -20 mA/cm^2**	**40.5**	**11.8**	**7.9**	**39.8**	**3.43**

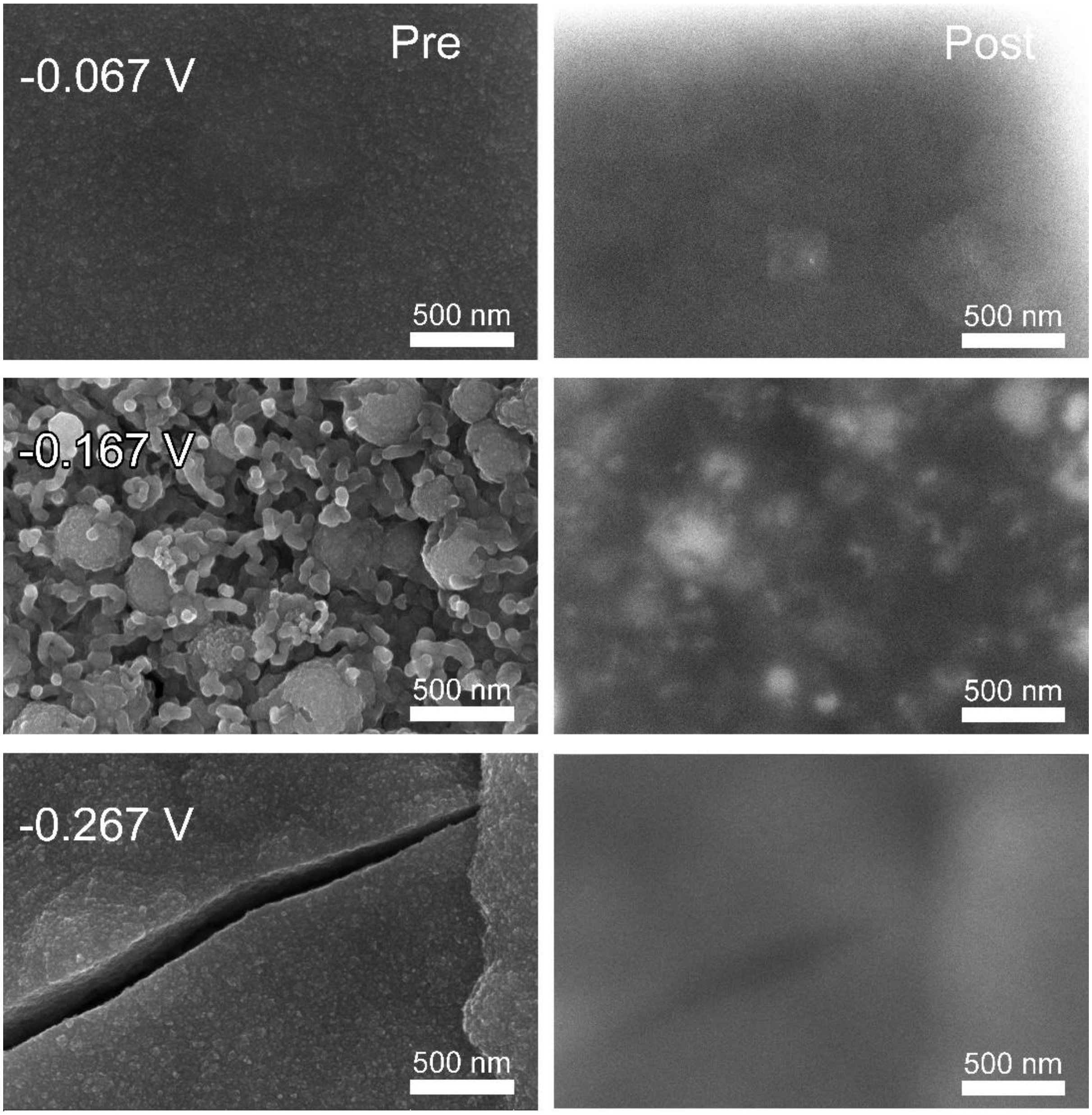

Figure S4. SEM micrographs of Ni-Mo/StSt before (left) and after (right) chrono-amperometric catalysis for 24 h in 1 M KOH at different potentials.

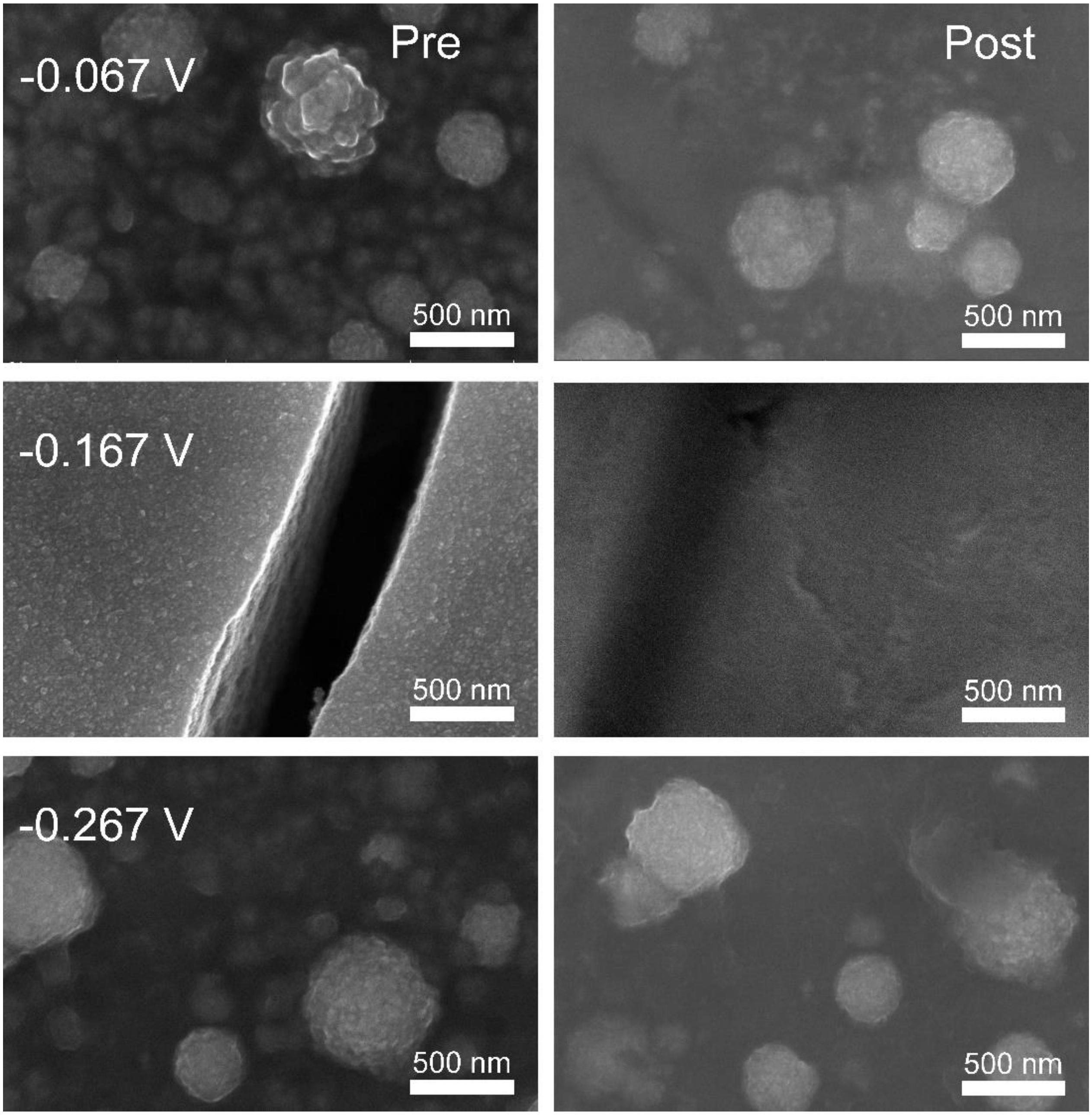

Figure S5. SEM micrographs of Ni-Mo/Ni before (left) and after (right) chrono-amperometric catalysis for 24 h in 1 M KOH at different potentials.

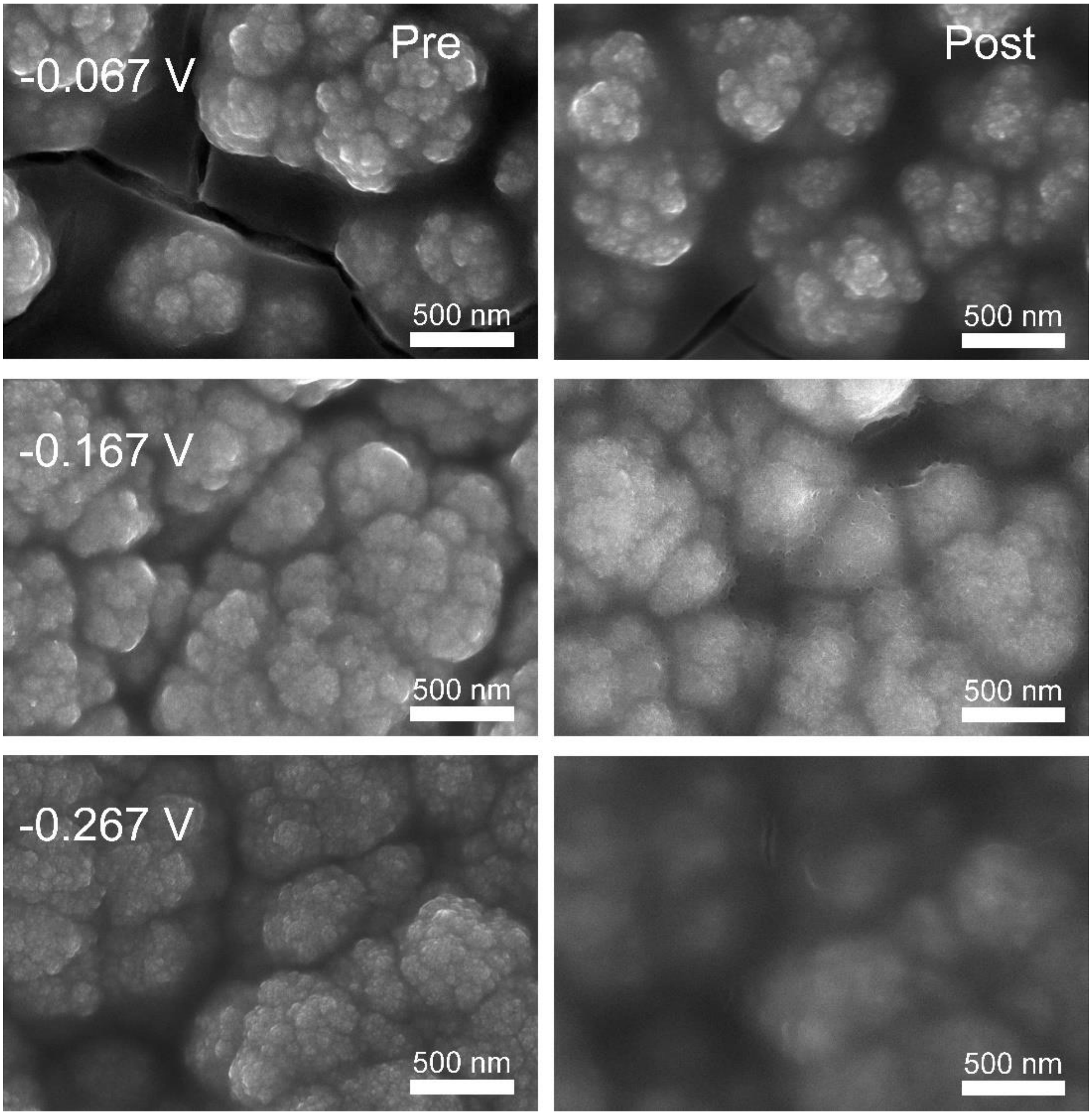

Figure S6. SEM micrographs of Ni-Mo/Cu before (left) and after (right) chrono-amperometric catalysis for 24 h in 1 M KOH at different potentials.

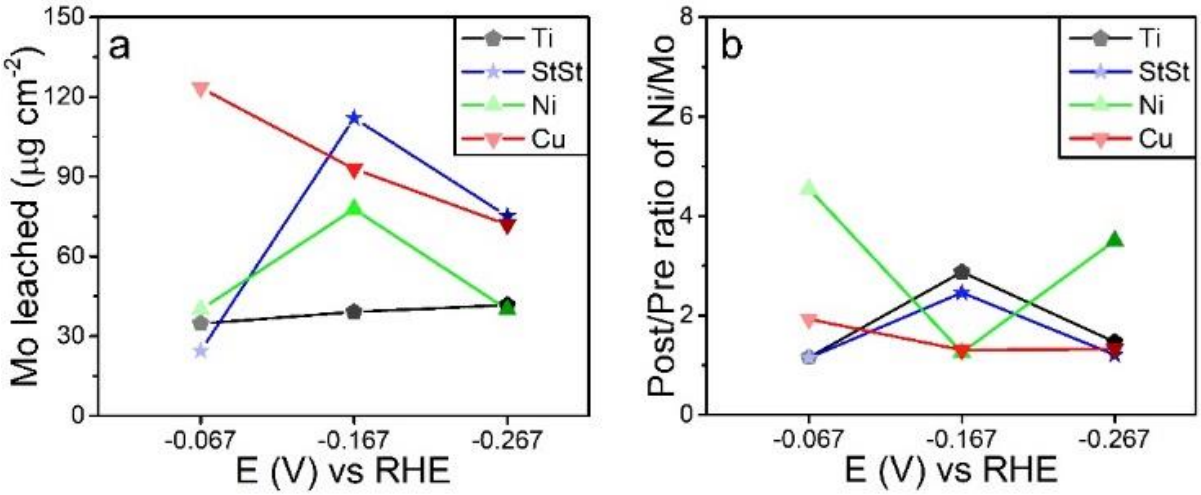

Figure S7. a) The amount of Mo leached as a function of applied potential after 24 h of chrono-amperometric catalysis on Ni-Mo on Ti (pentagons), StSt (stars), Ni (upward triangles), and Cu (downward triangles). b) The ratio of Ni/Mo ratios found by EDX before and after catalysis for the same materials.

Table S3. Atomic ratios as found by SEM-EDX before (regular) and after (bold) 24 h of chrono-amperometry. Only Ni, Mo, Na and K are considered and thus add up to 100 at%. Other elements are (adventitious) C, O, and Si, the latter being present as SiC particles that had left by the sanding paper. For Ni-Mo/StSt Fe and Cr were also observed, for Ni-Mo/Cu Cu was observed. The last column shows the ratio of Ni/Mo. It should be noted that the Ni/Mo ratio for Ni-Mo/Ni and Ni-Mo/StSt is not representative due to the substrate also giving off signal which is very dependent on the thickness.

	Ni (at%)	Mo (at%)	Na (at%)	K (at%)	Ni/Mo ratio
Ni-Mo/StSt -0.067 V	65.0	30.3	4.9	-	2.15
**Ni-Mo/StSt -0.067 V**	**68.8**	**27.9**	**-**	**3.3**	**2.47**
Ni-Mo/StSt -0.167 V	69.1	19.1	11.8	-	3.62
**Ni-Mo/StSt -0.167 V**	**73.2**	**8.2**	**-**	**18.6**	**8.90**
Ni-Mo/StSt -0.267 V	68.5	29.6	1.9	-	2.31
**Ni-Mo/StSt -0.267 V**	**70.0**	**25.1**	**-**	**4.9**	**2.79**
Ni-Mo/Ni -0.067 V	81.5	11.8	6.7	-	6.91
**Ni-Mo/Ni -0.067 V**	**94.3**	**3**	**-**	**2.7**	**31.43**
Ni-Mo/Ni -0.167 V	65.5	31	3.5	-	2.11
**Ni-Mo/Ni -0.167 V**	**70.7**	**26.7**	**-**	**2.6**	**2.65**
Ni-Mo/Ni -0.267 V	87.5	3.8	8.7	-	23.03
**Ni-Mo/Ni -0.267 V**	**96.9**	**1.2**	**-**	**1.9**	**80.75**
Ni-Mo/Cu -0.067 V	63.2	27	9.8	-	2.34
**Ni-Mo/Cu -0.067 V**	**81.1**	**18.0**	**-**	**0.8**	**4.51**
Ni-Mo/Cu -0.167 V	72.9	24.9	2.1	-	2.93
**Ni-Mo/Cu -0.167 V**	**76.3**	**20.0**	**-**	**3.8**	**3.82**
Ni-Mo/Cu -0.267 V	66.5	27.5	6	-	2.42
**Ni-Mo/Cu -0.267 V**	**72.0**	**22.4**	**0.3**	**5.2**	**3.21**

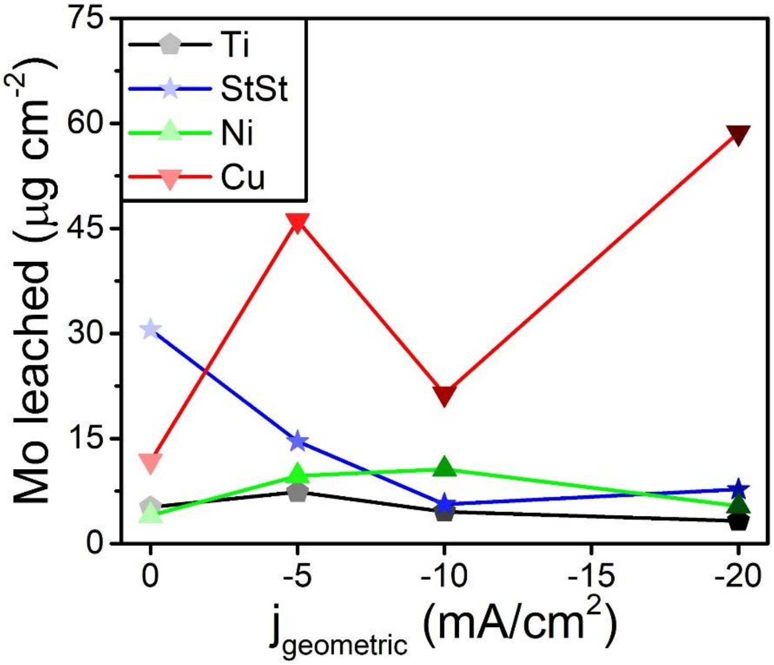

Figure S8. ICP-AES signal of Mo leaching after the *in situ* UV-Vis spectroscopy experiments as a function of applied current for 4 h on Ni-Mo at different substrates: Ti (pentagons), StSt (stars), Ni (upward triangles) and Cu (downward triangles).

Table S4. Atomic ratios as found by SEM-EDX before (regular) and after (bold) the *in situ* UV-Vis spectroscopy experiments. Only Ni, Mo, Na and K are considered and thus add up to 100 at%. Other elements that were observed are (adventitious) C, O, and Si, the latter sometimes being present as SiC particles that had been stuck in groves left by the sanding paper. For Ni-Mo/StSt Fe and Cr were also observed, for Ni-Mo/Cu Cu was observed. The last column shows the ratio of Ni/Mo. It should be noted that the Ni/Mo ratio for Ni-Mo/Ni and Ni-Mo/StSt is not representative due to the substrate also giving off signal which is very dependent on the thickness.

	Ni (at%)	Mo (at%)	Na (at%)	K (at%)	Ni/Mo ratio
Ni-Mo/StSt 0 mA/cm^2	66.1	31.1	2.9	-	2.13
**Ni-Mo/StSt 0 mA/cm^2**	**61.9**	**32.0**	**2.7**	**3.4**	**1.93**
Ni-Mo/StSt -5 mA/cm^2	63.1	34.4	2.5	-	1.83
**Ni-Mo/StSt -5 mA/cm^2**	**65.6**	**31.3**	**-**	**3.2**	**2.10**
Ni-Mo/StSt -10 mA/cm^2	71.0	19.0	10	-	3.74
**Ni-Mo/StSt -10 mA/cm^2**	**86.0**	**11.6**	**-**	**1.9**	**7.41**
Ni-Mo/StSt -20 mA/cm^2	65.3	22.5	12.2	-	2.90
**Ni-Mo/StSt -20 mA/cm^2**	**74.7**	**22.7**	**-**	**2.6**	**3.29**
Ni-Mo/Ni 0 mA/cm^2	68.9	28.8	2.3	-	2.39
**Ni-Mo/Ni 0 mA/cm^2**	**65.0**	**30.9**	**2.0**	**2.2**	**2.10**
Ni-Mo/Ni -5 mA/cm^2	79.2	18.9	1.9	-	4.19
**Ni-Mo/Ni -5 mA/cm^2**	**77.9**	**18.3**	**2.1**	**1.7**	**4.26**
Ni-Mo/Ni -10 mA/cm^2	68.8	22.2	9	-	3.10
**Ni-Mo/Ni -10 mA/cm^2**	**67.5**	**24.1**	**1.7**	**6.8**	**2.80**
Ni-Mo/Ni -20 mA/cm^2	69.9	28.2	1.9	-	2.48
**Ni-Mo/Ni -20 mA/cm^2**	**67.1**	**27.9**	**1.1**	**4**	**2.41**
Ni-Mo/Cu 0 mA/cm^2	67	27.2	5.8	-	2.46
**Ni-Mo/ Cu 0 mA/cm^2**	**73.2**	**26.7**	**-**	**0.1**	**2.74**
Ni-Mo/Cu -5 mA/cm^2	62.8	28.6	8.5	-	2.20
**Ni-Mo/Cu -5 mA/cm^2**	**69**	**24.9**	**-**	**6.1**	**2.77**
Ni-Mo/Cu -10 mA/cm^2	68.5	28.3	3.2	-	2.42
**Ni-Mo/Cu -10 mA/cm^2**	**67.4**	**22.9**	**-**	**9.7**	**2.94**
Ni-Mo/Cu -20 mA/cm^2	68.3	29.3	2.4	-	2.33
**Ni-Mo/Cu -20 mA/cm^2**	**70.0**	**26.7**	**-**	**3.4**	**2.62**

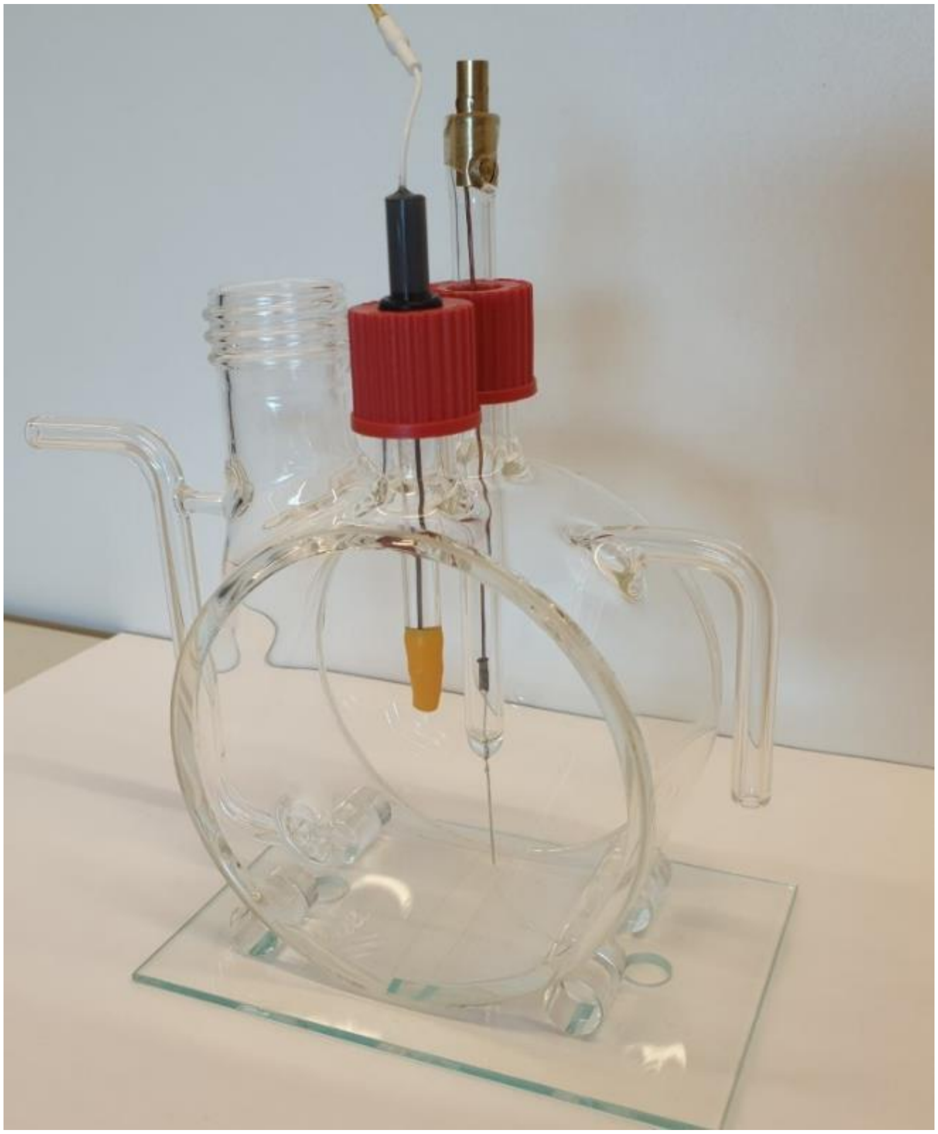

Figure S9. Photograph of the *in situ* UV-Vis spectroscopy cell with the Pt wire and BASi reference electrode.

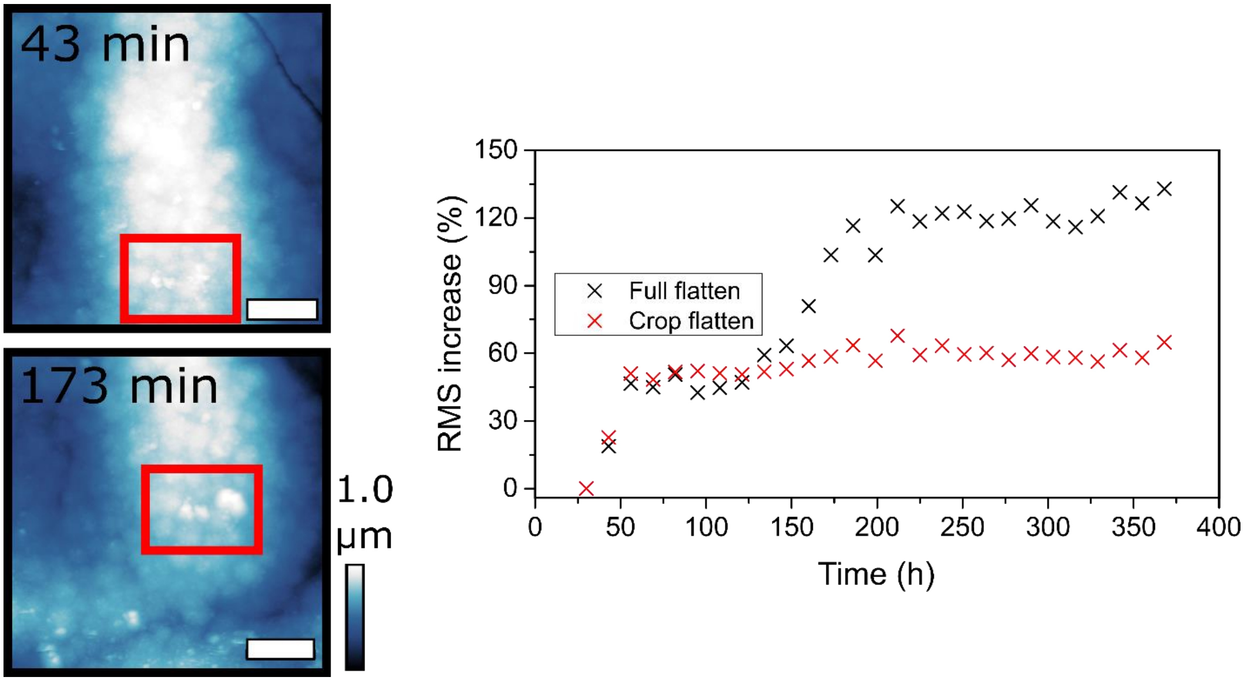

Figure S10. AFM micrographs of Ni-Mo-Ti in 6 M solution at different times, showing the displacement due to drift. The black box represents the full image flattening area, while the red box represents the cropped selection area. Right: The RMS of the selected area (red) is plotted over time when flattening is performed over the red box only, or the full black box.
